# Sociological Accounts of Donor Siblings’ Experiences: Their Importance for Self-Identity and New Kinship Relations

**DOI:** 10.3390/ijerph19042002

**Published:** 2022-02-11

**Authors:** Rosanna Hertz

**Affiliations:** Departments of Sociology and Women’s and Gender Studies, Wellesley College, Wellesley, MA 02481, USA; rhertz@wellesley.edu

**Keywords:** new kinship, donor-linked families, donor-siblings, siblings, identity

## Abstract

A fundamental part of the adolescent self is formed through interaction with intimates, usually family members whose roles are reasonably well-defined. However, donor siblings—children who share a sperm donor—lack social scripts for interacting with one another, particularly when they are raised in different households. Moreover, they are often challenged to figure out their relationship to newly discovered genetic relatives. This article examines how donor-conceived teens and young adults navigate relationships with their half siblings and form intimate relationships. Drawing on Garfinkel’s concept of indexing, or the use of familiar categories to make sense of new situations, these youth rely upon their personal knowledge of friends and family to figure out what kinds of relationship they can develop with donor siblings. Based on interviews with 62 youth aged 14–28, who had their own social media accounts and who had chosen to establish contact with their donor siblings, the articles discusses the three stages most donor siblings go through—“anticipation”, “first contact”, and “relationship building”—and the way those stages shape individual identity formation. In the course of the analysis, the article also explores whether interaction with donor siblings affected individual’s sense of identity and whether feelings of closeness with donor siblings differ for youth raised as solo children versus those who have siblings with whom they share a household. For those who report feeling close, youth describe how intimacy is sustained and negotiated at a distance. Finally, as much as youth pick and choose intimates from their larger network, the article also argues that their collective identity as related-kin members remains.

## 1. Introduction

Children conceived from donor gametes are born into nuclear families, but they are also born into an invisible (and potentially quite large) group of half-siblings who share half their genetic traits and also share deep questions about the roots of their own identity. Scholars of new kinship studies have been particularly focused on the ways in which new reproductive technologies ground families in bio-genetic connections while also presuming that these connections could constitute family ties. These scholars have shown how the cultural narrative around nuclear families has been stretched to accommodate children conceived through new reproductive technologies (see, for example, [1,2,3,4]). Moreover, research has documented various aspects of donor-conception on identity formation including the influence of different types of families on donor-conceived adolescents (see, for example, [5,6,7]); parental facilitation of children’s understanding of donor conception [8] and the request for information by donor-conceived individuals about their donors [9].

Still to be explored, however, are important questions about donor siblings, family ties, and individual identity. For example, while donor-conception anchors their identity in a genetic grounding of kinship, donor-conceived individuals must contend with the fact that part of their identity comes from an anonymous person whose identity is protected and who they may never be able meet. (In the U.S. context donors can still remain forever anonymous. Donor-conceived individuals with identity-release donors and open donors can contact donors when they reach age 18. By contrast, establishing contact with families who happened to purchase the same sperm donor is not age-related.) Children may reside in a nuclear family, but how does an anonymous genetic donor figure into a child’s search for identity from their genetic half-siblings? Moreover, the discovery of donor siblings could offer insight into shared, genetically rooted traits, but donor siblings have no claim to kinship. How do donor siblings fit into the cultural narrative of the nuclear family? Equally interesting, how might children raised with siblings in their nuclear families feel about becoming close to their donor siblings in comparison to those who are raised as solo (“only”) children? Contrasting the experiences of donor-conceived youth that have grown up in different social environments may help us unravel the differences between social and biological components of kinship.

Voluntary registries, ancestry searches, and more openness have also increased awareness of the possibility of locating half-siblings and forming relationships between donor-conceived offspring. Social media has dramatically enhanced the ability of individuals who share the same sperm donor to find one another online [10]. At the same time, a broad cultural turn toward believing that genes are important to a child’s identity (see [10,11,12]), has encouraged parents to seek more insight into their child’s genetic origins through connecting with their child’s half-siblings [13,14].

In her theorizing about the role of in vitro fertilization in redefining life, technology, and biology, Sarah Franklin [15] (p. 34) introduced the concept of “biological relativity” to indicate how assisted reproductive technologies have played a significant role in reproducing familiar narratives, such as the universal desire for parenthood, while simultaneously blurring the boundaries of heteronormative parenthood through gamete donation and artificial insemination. Though Franklin provocatively amplified how “biological relatedness” is enabling new biological relatives, these “new relatives” have until recently remained in the shadows and were rarely studied. Scholars find that the use of anonymous gamete donors and Internet search tools are giving rise to a whole new category of kin [2,11,16,17]. These offspring are close genetic relatives (as half “siblings”) who are being raised by other parents and who need to be discovered. Moreover, like adoptees, donor-conceived individuals face questions that are quite distinct from their parents’ experiences and understanding of kinship. In short, genetic relatedness to multiple half-siblings has created a dynamic environment for which there is no map to aid in navigation.

Encounters with genetic relatives carry significant implications for identity and self-concept among children, as well as for the meaning of kinship more generally. A fundamental part of the adolescent self is formed through interaction with intimates (e.g., parents and siblings) whose roles are reasonably well-defined [18,19,20]. However, children who share a sperm donor but who are raised in different households face unprecedented questions: does the discovery of close genetic relatives play a role in the formation of a child’s identity and self-concept? Is it possible for youth to develop intimacy or to feel close to their donor siblings on par with others who live in close proximity? Do relations among donor siblings differ from—or even compete with—relations with siblings from the same household when it comes to shaping the self?

To date, research on donor-linked children and families has not provided answers to those questions. In particular, donor-conceived youth have rarely been asked their views on the implications of their newly found relatedness (for recent exceptions see [17,21,22]). Overall, those studies that focus on donor conceived individuals report mostly positive experiences especially around contributing to a sense of identity [23,24,25]. The biggest research insights have had to do with the proliferation and differentiation of networks of donor-linked offspring and a sense of “family connection” [10,17,26]. Hertz and Nelson [17], for example, have shown that while not all parents (or children) show interest in the existence of donor siblings, many enthusiastically seek out connections—often with the hope that more information about their child’s genetic relatives will fill gaps in their knowledge of genetic predispositions and, in some cases, contribute to a child’s stock of social capital through richer networks [17]. Andreassen [27] and Indekeu et al. [22] have explored the experience of relatedness and the possibility of intimacy in donor-linked networks with mixed results. Andreassen [27] interviewed mothers in a donor-linked Facebook group from Denmark, Sweden, and Norway and found that deep connections were formed among parents around similarities with their donor-linked children. Indekeu et al. [22] interviewed adult donor-conceived members of five networks through the Dutch organization, Fiom, and found that the instability of those networks made it difficult to experience intimacy with more than a few individual members, despite feelings of group relatedness. While new members were welcomed by some of the already present members, the continuously expanding membership made it challenging to constantly readjust closeness or distance.

This article uses interviews with donor-linked teens and young adults to explore how they navigate relationships with their half siblings and how they situate siblinghood within the context of other relationships, real or imagined, that already exist in their lives. Social and cultural contexts are important for two reasons. First, conventional notions of brothers and sisters shape children’s expectations about what relationships with donor siblings could or ought to be. Second, persistent differences between what those conventions proscribe and what youth experience could prompt the creation of new understandings.

## 2. Donor Siblings in Context

Donor-conceived individuals reside in a context bordered by convention and novelty. On one side, labels such as brother, sister, or cousin imply a great deal about such things as familiarity, intimacy, and reciprocal obligations. (“Donor siblings” with the shorthand “diblings” [17] and “same-donor peers” [21] are terms commonly used to refer to others who share a genetic tie.) On the other side, even if parents have been open about their use of a donor, few children are prepared to comprehend what it means to have one or more (or many) half-siblings who live in other households. In the case of children raised in a household with other siblings (e.g., blended families, families with a mix of donor-conceived and parentally conceived children or siblings who might share the same or different donors), it is not immediately obvious who is closer: a sibling with whom a household is shared or one with whom genes are shared but not other indicators of family.

A similar tension attaches to the influence siblings have on the formation of a child’s identity or self-concept. For example, Davies [20] found that interactions with siblings who reside in the same household played an important role in shaping a young person’s sense of self. In fact, Davies’ [20] (p. 10) interviewees described their identities largely through statements of similarity and difference, competition and friendliness with their siblings. However, donor siblings are embedded in a different “ecological context”—to borrow from McGuire and Shanahan [28] (p. 73)—and correlates of siblinghood such as intimacy and shared understanding cannot be taken for granted. Neither can their effects on identity and self-concepts.

For these reasons, it is important to pay close attention to the social and cultural contexts surrounding individual and group identity. In the next section, I focus on how donor siblings themselves made sense of one another. In subsequent sections, I use the interviews to provide deeper insight into the evolution of donor sibling relationships.

## 3. The Study and Methods

Data for this article were originally collected as part of a larger study of donor-conceived families and network formation funded by N.S.F. during the years of 2014–2017, designed to examine donor-use and the search for donor siblings. The study consisted of in-depth interviews with 212 parents and their 154 donor-conceived children, as well as 12 donors related to these children. All parents had told their children they were donor- conceived. In this sample, parents had purchased gametes from multiple sperm banks and they (or their children) had located their half-siblings through donor sibling registries. Participants were interviewed in parts of the U.S. associated historically with the rise of the fertility industry as big business. We traveled to six major metropolitan areas within the U.S. to conduct in-person interviews. Parents and their donor-conceived children were interviewed separately, usually simultaneously. This allowed each person to tell their own account of events and relationships. The majority of interviews were conducted in-person. When it was not possible to meet face-to-face, interviews were conducted over Skype. (Of the 154 interviews with children ages 10–28, 39 children were interviewed on Skype. Of the 212 parents, 53 were interviewed on Skype.) Interviewing in person or virtually produced the same quality of data [17]). (See Hertz and Nelson, [17] (pp. 225–241) for further methodological details related to data collection.)

Semi-structured questions with probes were developed and parents and children were asked similar questions (see Hertz and Nelson [17] for strategies used to interview donor-conceived children of different ages). Donor-conceived children (ages 10–28) were told that we would not discuss what they said with their parents and that they were free to talk about the interview with them if they wanted. When we interviewed families within the same network, we were careful not to discuss interviews we had already conducted with other individuals within their network. Interviews lasted between 1 and 2 hours.

All interviews were fully transcribed and all respondents were given pseudonyms. We maintained contact through holiday cards, often people wrote back sharing updates. Interviews were originally coded using HyperResearch. We developed a coding scheme using both broad codes (such as donor sibling, donor, relations with parents, birth stories) and then more specific codes (e.g., subcodes within donor siblings include feelings about learning about donor siblings, first meetings, development of relationships with donor siblings, forms of communication). Construction of the codes was guided by the principles of grounded theory, with emerging themes identified and then reanalyzed for consistency and completeness [29,30]. 

The analysis presented in this paper is based upon a subset of interviews with 62 youth aged 14–28 (median age 17.5), who had their own social media accounts and who had chosen to establish and maintain contact with their donor siblings. Among the respondents 36 were girls (median age 17) and 26 were boys (median age 18). The majority of youth were white, identified as heterosexual, and thought their families were middle class. At the time of these interviews, 17 youth had contact with their donors and 45 youth did not. Those who had made contact usually had identity release donors. Members within each donor network lived throughout the U.S. and some lived abroad, making it difficult to meet regularly in person. Parents of these youth provided information about their household incomes, and 43% earned below USD 100,000. Youth often noted that they would have liked to meet more frequently with their donor siblings, but family resources did not always permit this.

In 2021, these 62 interviews were recoded by myself and a research assistant to ensure intercoder reliability. The new coding scheme examined how they viewed their donor siblings along several dimensions: in comparison to siblings in their household; in comparison to friends from school or camp; in terms of how they were described to others (e.g., half-brother/sister, cousin or other); feeling of intimacy or distance; and the content of their communication. The tables presented in this article were constructed based upon recoding of the initial interviews.

Ten follow-up interviews with current college-age youth were conducted in 2020–2021 in order to learn about changes in their donor sibling relationships, about how these relationships continued without their parent’s supervision and whether feelings of closeness change when additional donor siblings join. On Zoom, individual interviews were conducted with four youth who have siblings in their households and four youth who do not have household siblings. These interviews were meant to flesh out siblinghood understandings and to confirm my recoding process. Finally, a pair of donor siblings from a large network that I have been following were interviewed together, and they provide a current look at the continued functioning of the broader network. Lack of funding limited my ability to continue to gather additional updates. I indicate throughout when I am referring to a follow-up interview.

This article draws upon original data that were not analyzed prior to comparing youth with household and non-household siblings. Moreover, the themes developed in this article resulted from recoding this subset of interviews.

## 4. Results

Table 1 presents youth responses to questions about initiating contact with their donor siblings. Consistent with earlier research that describes parent initiated searches [9,11,13], this table highlights that regardless of family type, parents were most likely to take the first steps toward contacting other donor-conceived families in their network, often without consulting with their children. After cautiously vetting families they located on registries, they told their children about their discovery of half-siblings [17]. Among these interviewees, there was more child-initiated contact when youth had same-sex parents. However, even these youth enlisted their parents’ help as they were too young to sign onto registries on their own. Moreover, regardless of family type, parents also arranged for in-person meetings, often with members of the larger donor network. As these youth entered their teen years they established their own relationships with their donor siblings.

Since parents in all family types were enthusiastic about facilitating contact with their child’s half siblings, these youth felt comfortable about how these donor siblings could become meaningful in their lives. Therefore, I wanted to understand how donor-conceived youth incorporate these genetic relatives into their lives; how donor siblings might be important to their identity; and whether these connections were like siblings raised together.

## 5. Making Sense of Unprecedented Relationships

When asked to recall their initial reaction to learning that there were kids “out there” with whom they shared a sperm donor, our interviewees expressed a wide array of emotions: surprise, confusion, and curiosity were among the most frequently mentioned. Oliver recalled asking: “just because they have the same genetics, does that mean they’re my family?” The fact of shared genes amplified interest in knowing more about peers who were otherwise strangers. Andy, a high school senior, described the sensation this way: “the fact that we share such similar blood is … a catalyst for getting to know each other”. He recalled trying to puzzle through the situation as an 8-year-old: “the toughest part was when I don’t know what questions to ask. Like you don’t know how touchy you can be on a certain subject, how OK certain things are. Because these are your family, and if they were your normal siblings that you grew up with, this would be stuff you already shared. Stuff like political orientation or socioeconomic status”. Even those who ultimately chose not to make contact grappled early on to put a label on their donor siblings.

Unprecedented relationships are often socially scripted the first time using conventional ideas and experiences. Garfinkel [31] referred to this as “indexing”, a tactic for sorting things and people into categories we already know. According to Hertz and Nelson [17], youth such as Andy (quoted above) often started by borrowing from ideas that they already had about siblings—evidenced, for example by his use of the term “normal siblings”. Andy and his peers used familiar tropes about siblinghood to anticipate things they imagined siblings might bring, such as advice, companionship, and/or commiseration. They narrate their accounts of their donor siblings through these cultural understandings.

Donor siblings can also be a curiosity because they offer unique benefits. For example, Nelson et al. [32] (p. 32) found in their survey of 492 donor siblings: “to be sure, (donor) siblings provide access to, and a glimpse at paternal kin. But unlike sisters and brothers, who grew up together, these siblings are ‘perfect’, as one interviewee explained: i.e., related just to them and (not to their parents) and posing no immediate threat to parental love, resources, and time”.

In other words, they are imagined as “fun”, “cool”, and “neat”, and people who might immediately understand them. Equally important, half-siblings might give them insight into their genetic origins (such as shared physical traits and interests) at a moment in time when they are engaged in their own search for identity.

An important and recurrent theme in interviews with the youth who chose to get to know their donor siblings was their openness to new and potentially intimate relationships. No one foresaw their encounters as likely to displace or diminish the intimate relationships already in their lives. In making this point, I draw upon Jamieson’s [33] (p. 1) definition of intimacy and intimate relationships: “Although there is no universal definition, intimate relationships are a type of personal relationships that are subjectively experienced and may be socially recognized as close…. Practices of intimacy … enable, generate and sustain a subjective sense of closeness”. In other words, the majority of interviewees took for granted that if they were going to reach out to their donor siblings intimacy of some kind would follow.

The reanalysis of these interviews for this article revealed that donor sibling interaction occurred in three distinct stages: anticipation, first contact, and relationship building. During these stages, kids experimented with using what was known and familiar to them in friendships and families to incorporate new people in their lives. Based upon the evolution of these interactions, indexing either “succeeded” by reaffirming conventional labels of friend or family or it “failed” in that it led them to create categories that were not strictly one or the other.

## 6. Anticipation

Prior to first contact, children (and often parents) tried to figure out what they wanted to accomplish from meeting. Youth had widely divergent expectations. For example, Gabriella, who had no siblings within her immediate family, hoped “that I would find maybe like a true brother or sister—like where you’re really close with them”. Diane, a 17 year-old high school student who still lived at home with a brother, put it this way: “I wanted an older sister who would lend me her clothes or give me her makeup. Something like you read about in the books. I wanted something a little more stereotypical in my life”. Ethan, who had two sisters, recalled hoping he would find a brother when he heard that his mom had planned a first meeting with his donor siblings: “maybe the guys in my donor-group would have something to talk about because I didn’t really have a whole lot of intimate guy friends and I wanted a brother. So I thought that might be cool”. By contrast, Marc who already had a brother and a step-sister “thought that donor siblings would be like a fun new friend group” but added that “I don’t really need more family than I already have”.

In anticipation of first contact, most of the youth recalled using definitions of friend versus family to set boundaries on their obligations and commitment to their donor siblings. Those who used the language of family anticipated more serious, intimate relationships to develop; friendship, on the other hand, connoted distance, allowing for fun and perhaps intimacy without the intensity or reciprocity that familial relationships imply. Later, in this article, I discuss the relationship between household composition, gender, and feelings of intimacy.

## 7. First Contact

Imagined relationships were challenged as youth first encountered the real people attached to their fantasies. Instead of using friendship and family as abstractions through which to assign types of obligation, they compared these new emotional connections to ones they felt for “real” friends and family.

As they interacted for the first time, many of the youth began experimenting with a siblinghood narrative grounded in similarities and differences, especially around physical resemblance and personality traits, much as non-donor-conceived children who grew up in the same household [20]. As these youth recognized themselves in their donor siblings, they began to feel that their donor siblings could become intimates. For instance, Sally (age 19) recalled the first time she met nine of her donor siblings: “I spent a lot of the time just staring at them in disbelief. I have a physical similarity to basically everyone in the group. I was just really consumed by the fact that these were siblings or I guess they were genetically related to me in a very close way… I spent a lot of time really just observing and looking and analyzing what was going on.”

The fact of physical resemblance encouraged Sally and others to reflect on the multiple bases of kinship in their lives. Sally explained: “I grew up with just my mother. I had one cousin that I was close to. I never really had much of a family except my mom’s sorority sisters (and they) have completely absorbed us into their family. I have 30 people that I call cousins, aunts, and uncles that I’m very close to and love and see often.”

The idea that kin could be chosen—not just given or imposed—made it possible to see donor siblings as potential kin without necessarily displacing household siblings raised together.

First contact also gave children the opportunity to use play to “try out” group and kinship definitions. For instance, at the first meeting of nine donor siblings ranging in age from 10–15, the older kids reported feeling protective and watchful of the younger ones as they scurried around late at night at the hotel where they were staying. Even though their ages were relatively close, they squeezed themselves into a hierarchy based on age differences. New joiners to the group embraced this hierarchy as a central feature of their relatedness. Similarly, Hertz and Nelson [17] (pp. 116–117) found that age and gender emerged as major bonding mechanisms among group members and as an organizational strategy for early network meetings or reunions. In effect, these teens used familial practices to align with their ideas about intimacy and siblinghood.

## 8. Relationship Building

Heavily influenced by first contact, relationships developed more fully in the building stage. Those who described their first encounters as positive, fun, or even deeply felt often concluded their meetings with promises to meet again. Unlike the promises to stay in touch made by children in summer camp in decades past, these young people had powerful tools such as video and text messaging at their fingertips that allowed them to connect immediately in order to continue their mutual explorations. For example, Beth and some of her donor siblings had to overcome the high cost of traveling to see one another: “the hardest part was that we had these new relationships and we had this new family that we loved… but we lived thousands of miles away. We can’t see each other all the time, even once a month or anything on a regular basis. It’s such a financial struggle to see each other because none of us are particularly well-off, though some more than others, of course… but we stay in touch through Snapchat, Facebook and texts”.

For those who were disappointed by their first meet up or felt only limited attachment, this stage may end with little change. Liza, age 19, felt that it was enough to simply have made contact: “it’s important for me to know them and to know that they are their own people and they exist in the world. I don’t have to like them, but they are still there and they’re not ideas of people, they are real human beings”.

Real relationship building required further face-to-face interactions and potentially the establishment of relationships with their donor sibling’s family, as well. Take the case of Jocelyn, who was 15 when she met her half sibling, Sam, who was a year younger. The monthly meet ups organized by their moms left Jocelyn and Sam little time alone. So, they decided to meet independently in order to establish a relationship. Jocelyn explained: “once I got my license, I would drive over and go visit him. We’d go get Fro-Yo or go to Chipotle. That, I think, actually helped our bond a lot, because we were able to talk without our parents there. We were actually able to talk like siblings… What links us? I think in high school especially, we had a really big sense of competition, because we’re both very smart. But he tried harder than I did. He just seemed so impressive, and I wanted to impress him. To be an older sister he could be proud of. That sense of wanting to make each other proud also helps to connect us”.

Jocelyn and Sam, who lived an hour and half away from each other, found mutual benefit in their relationship but admitted that it took time and work to establish.

During relationship building, we see a range of new narratives emerging. Some youth who shared genes were happy to leave it at that; donor siblings were no longer strangers but they failed to meet the test of either friendship or siblinghood. Others integrated donor offspring into a kinship narrative but, in the absence of something that made them interesting or attractive, donor siblings were accorded the status of distant relative: they existed but did warrant much attention. In addition, there were those who began to see siblinghood as more of an achieved status than an ascribed one. After a successful (and intentional) first connection, relationships can develop into something that resembles a social script about friendship or even shared family membership.

When indexing does not work—when, for example, the category does not do justice to the experience—new categories may come into being. However, new categories rarely survive without repetition; repetition requires dedicated effort, creativity and even courage. As I will show in the next section, developing a relationship with genetically related strangers involves bridging categories that are familiar but not always compatible.

## 9. Choosing Friends or Family

Youth who were raised with siblings and who had already developed strong understandings of siblinghood tended to set a high bar for their new genetic relatives. Almost three-quarters (71.4%) considered only those siblings in their household as immediate family (see Table 2). Alice, a high school senior, compared the feelings invoked by donor siblings to the feelings she had for the sister with whom she grew up, “Cydney is my sister. She is someone I know, who supported me throughout my life and who I feel I can talk to about things. The donor siblings don’t feel like siblings at all. They’re just people who happen to be related to me”. Alice was among the three-fourths of interviewees who distinguished between “true siblings” like Cydney and genetic relatives.

By contrast, youth raised as solo or only children were more likely to consider themselves close to one or more donor siblings (70.6% vs. 64.3%, see Table 2). Even more telling, solo youth were twice as likely to regard donor siblings as “immediate family” when compared to youth who had siblings in their household. In many instances, those raised as only children saw donor siblings as fulfillment of a wish they had earlier expressed to parents about wanting a sibling. Kyle, a 16 year old, put it this way: “I was excited that there’s finally siblings that I could have, that I’ve always wanted”.

Although there is a clear difference in reports of feeling close in regarding donor siblings as immediate family, it is important to note that nearly 70% of all interviewees depicted relationships with at least one donor sibling to be significant and meaningful. In other words, “donor sibling” had come to connote a level of emotional connection that had not existed previously. That level of connection did not apply to all donor siblings and it was not uniform. However, it did exist and it was explicitly called out by the overwhelming majority of the youth we interviewed. Usually siblings raised together agreed that they felt close to the same donor sibling(s). 

That did not mean that siblinghood came naturally. For example, Amy said: “I don’t even have a lot of boy cousins and I obviously don’t have any siblings so I never had a brother relationship besides my really good guy friends. So it was really hard to try and establish that brother-sister relationship”. Max recalled vividly being thrust into the role of “older brother” when he met the first of his many donor siblings, “Harper hates pigeons. It’s awful, to the point in which the first time we saw pigeons, which are everywhere in New York, and I mean everywhere, he came and hid behind me and was terrified. I’m all like, what is happening? I’m really confused about this but I learned that this person, who had just met me, was already seeking security from me”. Feeling brotherly or sisterly sometimes required a presentation of oneself that allowed for differences, such as annoying quirks or just not sharing the same viewpoints. For example, Sam was raised as an only child, and in his first year of college he talked about the distinctive qualities of his relationship with his donor sibling: “We do have a brother-sister relationship. I think you have to have that certain feeling of familiarity and closeness to be able to argue about certain things or to share our different opinions about things. Sometimes when I tell people my sister is such a sorority girl I think the way I say it is more loving at the same time, just in that brotherly, like, ‘Oh man, my sister. I don’t know what she’s doing’”.

In other words, solo children such as Amy, Max, and Sam found themselves “doing” siblinghood without any preparation. They had to figure out a social script that they had not grown up with as a natural part of their everyday family life.

The most interesting instances involved children who found themselves in a hybrid territory where donor siblings were neither “just friends” nor “immediate family”. Oliver explained that he liked all five kids he met but he considered only two to be family. Those two kids were not family because they shared the same donor but because they had “*become* family” (emphasis added)—similar to his “closest best friends” who were “random people I basically met and they became close enough to me that they crossed that same bridge”. The notion of a *bridge* is important because he felt obligated to the two high school friends he had selected to “crossover” from friends to family. He characterized this as a conscious process: “I had months of kind of being able to let myself say, ‘Listen, you need to be able to dismiss this idea of what is traditionally family’”. Donor siblings would never cross this bridge if these interviewees weren’t looking for (or weren’t prepared for) intimacy.

There were no significant gender-based differences in feelings of intimacy with donor siblings. In Table 3, we see that boys were as likely to say they were close to one or more donor siblings as girls (69.2% and 66.7%, respectively). Among those who had siblings in their nuclear families, boys and girls were equally likely to say that only the siblings they were growing up with in their household are immediate family (70% and 72.2%, respectively). Moreover, while boys and girls might have imagined donor siblings as providing a “brother” or “sister”, gender did not seem to influence decisions about who they ultimately become close to.

## 10. Intimacy at a Distance

Whether their encounters were casual or intense, these young people came to recognize that maintaining a relationship took real effort and dedication. Absent physical proximity in a household or a shared neighborhood and school, their paths did not cross frequently. In order to move from shared genes as a curiosity to friendship or something approximating feeling brotherly/sisterly kids had to work on siblinghood at a distance.

Interviews with these respondents revealed that donor offspring leveraged technology in their pursuit of siblinghood. The salience of technology lies in the choice it provides participants as to how open and frequent communication would be [34,35]. Intimacy is remade by these new genetic connectivities. 

Youth employed apps such as Instagram and Snapchat to initiate and sustain trusting relationships even in the absence of calling or meeting physically. This research extends Andreassen’s [27] (p. 367) important point that digital relationships “dissolve the traditional opposition between proximity and distance in relation to intimacy”. That is, intimacy and closeness are possible without sharing daily life.

In Table 4 we see that those who use social media were more likely to connect with individuals rather than engage in group chats. In addition, they were more likely to feel close to a donor sibling than those who engage in contact with subgroups or their entire group, such as on Facebook (data not shown). For example, Paul, age 16, and Isabel, age 19, text frequently and are extremely close. According to Paul, who trusts Isabel: “if I’m feeling I need someone to talk to, Isabel is there for me”. Isabel, who in her words feels a “strong protective instinct” for Paul, appreciated technology as it allowed her to advise and talk with Paul as well as stay aware of what was happening in Paul’s life. This “instinct” also distinguished Paul from the many other friends with whom she texted routinely.

Girls were more likely to maintain relationships through social media than boys (see Table 4). We don’t know if this meant that girls’ relationships flourished more because they were in social media contact. Boys said in the interviews that they picked up where they left off when they met in person. As Ethan (recently interviewed for the second time at age 21) explained: “I am not into social media and that might be atypical. But I don’t feel it matters for my feeling close to her. You know where it’s sort of where we cannot see each other for months, if not for a year and then we can come back together and things are perfectly amicable. We pick up where we left off”.

However, it is important to note that Ethan’s twin sister did maintain contact with both their favored donor sibling and their moms. Ethan said that he relied on his twin to be in communication and to pass along information.

I looked at whether length of time knowing donor siblings mattered to feeling close (data not shown) and found that the time-based determination of how close a respondent felt to a donor sibling hinged on knowing them for at least a year. In a follow up interview Julia, now age 21, talked about a new donor sibling. They remained in the early stages of trying to get to know each other. Looking at her Instagram profile, Julia “got a good idea of who she was and then I chose a topic to talk to her about”. Early on they identified a shared interest in a band and were able to “start a conversation based on that”. Even though they texted frequently, Julia’s emotional bond remained underdeveloped as compared to her longer-term relationship with her closest donor sibling. For now, they lean into the shared topic but have not established the complexities of sharing other intimate life details, nor have they met in person.

Finally, technology allowed young people to set clear and explicit boundaries to their relationships. For example, it made it possible to learn about new donor siblings as soon as they appeared. It offered people greater latitude in how they structured their communication, what they wanted to get out of it, and when they wanted to partake. While I have no evidence that technology accelerates the development of emotional bonds, it did allow people to do the work of siblinghood over long distances.

## 11. Collective Identity in the Network

The opportunities for bonding were not as great in “mature” networks (defined as those over five years old), particularly when those who joined early felt closer to the original core members (see also [22]). Sally explained: “if I spent more time with the others, I’m sure they would be elevated to the level of closeness I have with the first ones I met, but it has to do with the relationship that I’ve created with those four that’s really become family to me…we are there for each other in times of trouble”. Sally told us that as much as she felt related to everyone, she regarded as close siblings only the first four “half-siblings from my donor father”.

However, many networks remained meaningful precisely because members shared a donor whose identity was at least indirectly linked to their own. Even if they scoffed at the idea that genes determined identity, most members were at least curious to learn more about the donor (such as updates on health information, updates on what they wrote on their profile, or if they guessed correctly on how closely they resembled the donor). In instances where donors had not agreed to reveal themselves, network members had to figure out whether to search (or sleuth, as the activity was frequently called) and how broadly to share the information they uncovered. In the case of identity-release donors, when the oldest child turned age 18 they were often expected to post the letter they received from their shared donor to the network’s Facebook page. This gave donor siblings more information and some assurance that if they wrote to their donor they would write back or meet with them. This sense of obligation to the network reinforced a collective identity.

A useful example of how this operated came from interviews with 16 members of a network that identified itself as the “7008ers” [17] (pp. 105–135). In the early days of the 7008ers, there was no consensus as to how much information or communication individual members wanted if they could find their shared anonymous donor. Later in 2020, I learned that several members who wanted to locate their donor had found him through his family on a DNA registry. They also had polled the network before proceeding. When I met with Jenna and Nick, two of the closest donor siblings, for an update on their network, I recalled that these two members were among those in their network who felt that not knowing their donor left a “void” or “just a sort of mystery” and who wanted “closure which could only be reached by finding the real person”. In 2020, another subgroup did not feel that connecting (and meeting in person) with their anonymous donor was important. Others felt less certain, only wanting updated information. Feelings about the importance of connecting with their donor did not completely intersect with whom they felt closest with within their network. Jenna, one of those keen to identify the donor, explained: “when we started looking in earnest for our donor, we tried to do our absolute due diligence in making sure that everyone was respected. I reached out to each individual person, ‘If we find him, what’s your comfort level? Do you want nothing? Health information? Do you want name and face, nothing else? Do you want to meet him?’” Their sensitivity to sharing information also meant that they would not share personal information about their donor siblings with their donor.

In this regard, Jenna and Nick were not looking to include their donor in their existing network. According to Nick: “‘7008’—our name and our donor number—is us, the donor sibling network. We’ve kind of embodied that number for our group. And he’s just this new thing that’s not the center of the group, not peripheral, but not really a part of it. He’s kind of this other thing”.

In their view, the network was established prior to their donor reveal and existed separate from the relationships they might individually form with the donor. In effect, the donor might be the reason that these youth met, but his importance had diminished and their “sibling” relatedness, and closeness among subgroups, was independent of connecting with the donor.

## 12. Findings and Implications

One of the distinctive features of this research is its attention to the voices of the young people as they seek to make sense of a new category of kin, donor siblings. Rather than rely solely on the reports of parents, I explored the meanings that teens (and young adults) attached to their experiences. This approach has given us the opportunity to consider siblinghood as both a status and a process. The net benefit has been a rare opportunity to see a meaningful new social category unfold.

Three key findings deserve further consideration—for both future research and policy. First, donor siblings provide youth a resource for exploring the foundations of personal identity, i.e., the traits they share with others (that may be genetically-rooted) and the ones that are unique to the individual and their immediate context. They help youth develop a deeper understanding of what it means to be donor-conceived and to have an anonymous donor at a time when friends need to be educated, too, regardless of whether they can eventually meet him or receive more information about him. (Future research might want to explore how siblings raised together come to agree that they favored certain donor siblings within their network). Among these youth family type, geographic region or social class were not likely to factor into their decisions. However, though their involvement with donor siblings they did learn about families that were different than their own adding to their understanding of diversity (such as seeing a loving relationship between a same-sex couple that they did not experience in their single mom household.) While it is too soon to say whether interactions with donor siblings have an enduring impact on identity, it is nonetheless important to note that by the time they were interviewed most respondents had been in touch with their donor sibling network for several years.

Second, the feeling of belonging to a network of donor siblings (which might also include other children’s parents and conceivably the donor as well) turns out to be important even when membership is a weak tie. That is, membership in the network may be deemed purely instrumental (i.e., a source of potentially useful genetic or medical information only), but it is a membership nonetheless and a unique one by contrast to conventional kin ties. As we saw in a number of instances, membership in a network can be a doorway to deeper and more intimate social ties that supplement existing ones or that establish entirely new ones. Feelings of intimacy—whether based on blood, genes, or age—turn out to be far more real and durable than prior research had found. However, these youth did distinguish between obligations based upon relatedness to the entire network versus a smaller group with whom they formed more intimate ties.

Third, siblinghood takes work among youth who share a donor—a form of emotional labor largely taken for granted among siblings who share a common household. This is not to say that parents do not shape relations between siblings in a conventional household; it is to say that the benefits of having donor siblings cannot be taken for granted. They have to be intentionally established and sustained—in a fashion that siblings raised together discover only when they leave the household. These youth deploy ideas and concepts from familiar cultural tropes about people who are their friends or family to figure out how to situate their new half-siblings. Since these networks can grow quite large, youth do choose with whom they want to establish more intimate ties.

Future research would benefit from a longitudinal view of many of the findings and underlying processes described in this article. For example, it is not clear whether new members will find donor sibling networks as open to the formation of intimate ties as the originators did. In addition, on the policy side, the formal and legal status of donor-sibling ties remain unclear (see, for example, Cahn [2]). Donors in the U.S. are not legally obliged to identify themselves to offspring and sperm, egg, and embryo banks are largely unregulated, leaving important questions of data privacy and anonymity unresolved.

## Figures and Tables

**Table 1:** Who initiates contact with donor siblings.

Who Initiates Contact?		Family Type at Birth	
	Single Mother (*n* = 25)	Same-Sex Parents (*n* = 28)	Heterosexual Parents (*n* = 9)
Age at Interview (M years, range)	17.1, 15–21	19.1, 14–28	18.7, 16–25
Female	11 (44%)	20 (71.4%)	5 (55.6%)
Male	14 (56%)	8 (28.6%)	4 (44.4%)
Number of Siblings in House (M, range)	0.64, 0–3	0.82, 0–4	0.33 (0–1)
Parent Initiated	22 (88%)	19 (67.9%)	8 (88.9%)
Child Initiated	2 (8%)	9 (32.1%)	1 (11.1%)
Parent and Child Jointly Initiated	1 (4%)	0 (0%)	0 (0%)

**Table 2:** Siblings in the household and feelings about donor siblings.

Feelings about Donor Siblings	No Siblings in Household (*n* = 34)	Siblings in Household (*n* = 28)
Number of Siblings in House (M, range)	N/A	1.5, 1–4
Number of Donor Siblings (M, range) *	8.5, 1–41	11.8, 1–41
Intimacy with Donor Siblings		
Not close to donor siblings	10 (29.4%)	10 (35.7%)
Close to at least one donor sibling	24 (70.6%)	18 (64.3%)
Immediate Family Members		
Only considers siblings in household as immediate family	N/A	20 (71.4%)
Considers donor siblings to be immediate family	17 (50%)	7 (25.0%)
Did not respond	0	1 (3.6%)

* Includes only those that have revealed their identities. Others may be listed on registries but are unwilling to connect with donor siblings.

**Table 3:** Intimacy with donor siblings by gender.

Feelings about Donor Siblings	Gender	
	Male (*n* = 26)	Female (*n* = 36)
Intimacy with Donor Siblings		
Not close to donor siblings	8 (30.8%)	12 (33.3%)
Close to at least one donor sibling	18 (69.2%)	24 (66.7%)
Immediate Family Members *		
Only considers siblings in household as immediate family	7 (70%)	13 (72.2%)
Considers donor siblings/siblings in household as immediate family	3 (30%)	4 (22.2%)
Did not respond	0 (0%)	1 (5.6%)

* Only includes individuals who grew up with siblings in their households. For males, *n* = 10. For females, *n* = 18.

**Table 4:** Social media contact by gender.

Social Media Contact *	Gender	
	Male (*n* = 26)	Female (*n* = 36)
No Contact	4 (15.4%)	2 (5.6%)
Engages in contact with individuals (1)	18 (69.2%)	30 (83.3%)
Engages in contact with subgroups (2+)	2 (7.7%)	2 (5.6%)
Engages in contact only with entire group	2 (7.7%)	2 (5.6%)

* Facebook exchanges were excluded because Facebook was a platform primarily used by parents rather than the donor siblings themselves.

## Data Availability

Not applicable.
